# Supplementary figures and images for: Deer herbivory reduces web-building spider abundance by simplifying forest vegetation structure
Source: PeerJ. 2016 Sep 29;4:e2538. doi: 10.7717/peerj.2538 (PMC5047144; doi:10.7717/peerj.2538)

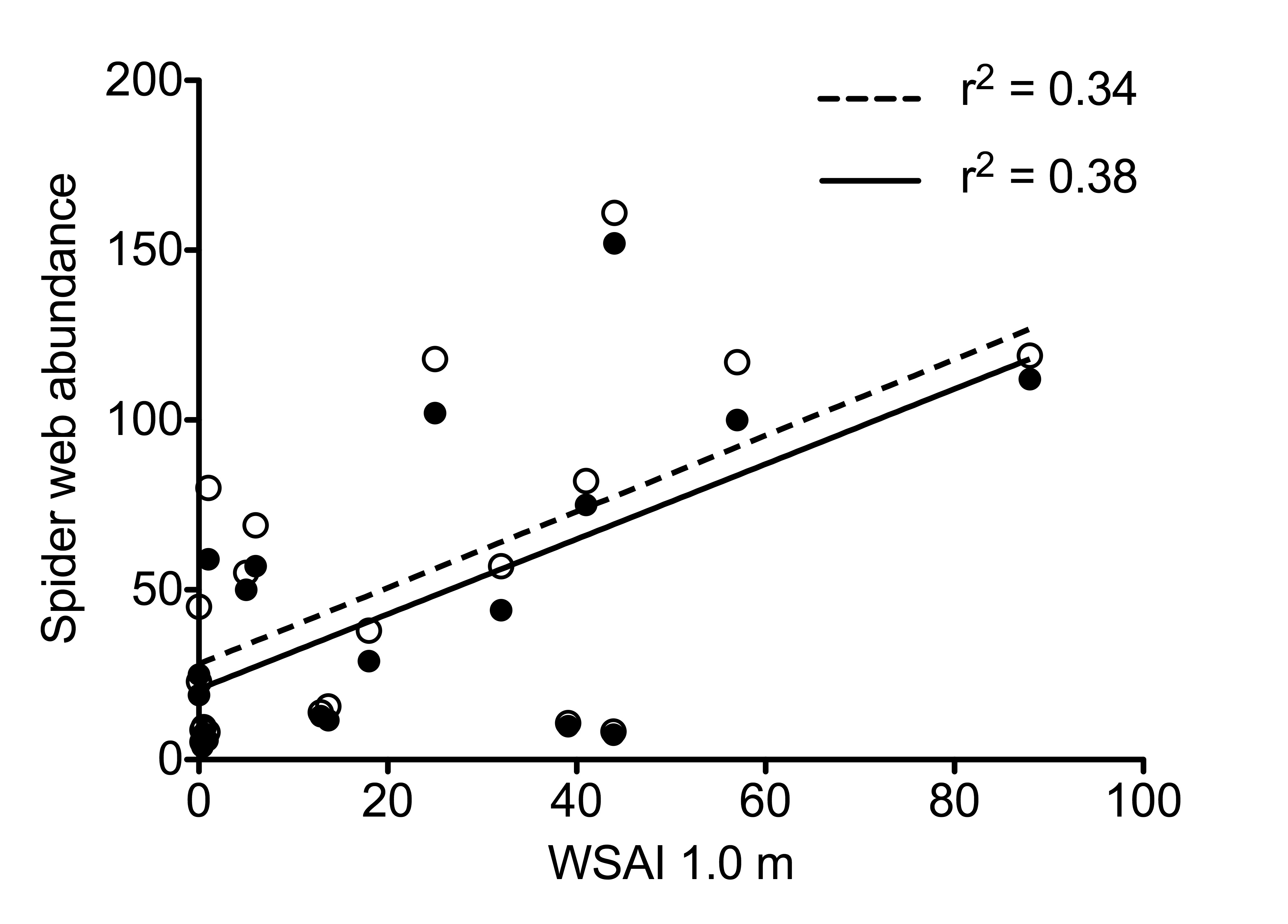

Supplement: Figure S1 — Spider web abundance as a function of WSAI with Agelenidae included (open circles, dashed line) and Agelenidae removed (dark circles, solid line). [file peerj-04-2538-s001.png]

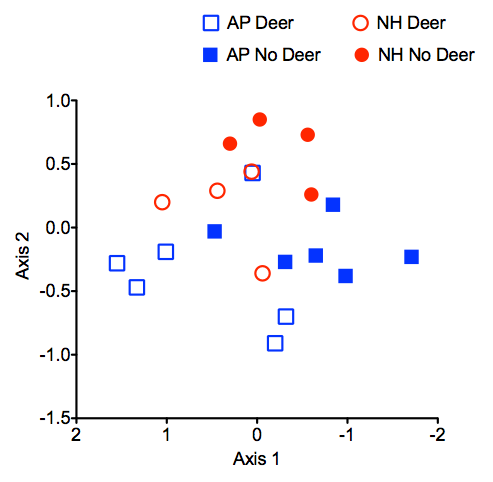

Supplement: Figure S2 — NMDS ordination with Agelenidae removed. The 3-dimensional solution had a Kruskal stress = 0.07; the two most variable dimensions are shown. [file peerj-04-2538-s002.png]
